# Growing Proficiency in Tic Disorder Care and Skills (GP-TiCS): a thematic analysis of patient and professional group experiences in primary care for primary tic disorders

**DOI:** 10.1186/s12875-026-03496-y

**Published:** 2026-07-30

**Authors:** Adam Parker, Blandine French, Madeleine J. Groom, Charlotte L. Hall

**Affiliations:** 1 Academic Unit of Mental Health & Clinical Neurosciences, School of Medicine, University of Nottingham, Nottingham, UK; 2 National Institute of Health and Care Research MindTech HealthTech Research Centre, Mental Health and Clinical Neurosciences, School of Medicine, University of Nottingham, Nottingham, UK; 3 National Institute of Health and Care Research Nottingham Biomedical Research Centre, University of Nottingham, Nottingham, UK; 4 School of Psychology, University of Nottingham, Nottingham, UK

**Keywords:** Tic disorders, Tourette syndrome, Experiences, Healthcare professionals, Primary care, Medical education, Knowledge, Stigma, Service provision

## Abstract

**Background:**

Around 300,000 people in the UK are thought to be living with Tourette syndrome (TS). Many people with tics and their families report negative experiences when approaching their general practitioner (GP) for advice and support. Limited education during medical training coupled with a lack of National Institute for Health and Care Excellence (NICE) guidelines for tic disorders (TD) creates clinical challenges for healthcare professionals (HCPs). Building on the results of an international systematic review of healthcare experiences for tics, this study aimed to further explore patient and professional experiences in primary care in England, specifically.

**Methods:**

A thematic analysis was conducted on interviews with adults with tics (*n* = 5), parents of children and young people (CYP) with tics (*n* = 5), GPs (*n* = 6), and secondary care HCPs working with people with tics (*n* = 4).

**Results:**

The *Factors Impacting GPs’ Knowledge and Education of Tics* theme suggested that limited education and guidance made it more difficult for GPs to support people presenting with tics yet also considers how surmounting pressures and expectations on primary care may exceed the responsibility and capabilities of GPs. *Inconsistent Availability of Support for Tics Across the Country* continues to be an issue for GPs making referrals to services and for people seeking support, while secondary HCPs report receiving unclear referrals with limited information which makes service allocation more difficult. The *Experiences of Primary Care for Tics* theme explores how the wider impact of living with tics is often left unexplored, while stigma, communication, and signposting were all identified areas for improvement.

**Conclusion:**

The results suggest accessible and appropriate education opportunities for GPs may improve experience for people presenting with tics by increasing GP knowledge and awareness of tics. This study precedes the development of an online learning resource for GPs.

**Supplementary Information:**

The online version contains supplementary material available at https://doi.org/10.1186/s12875-026-03496-y.

## Background

Primary tic disorders (TDs), including Tourette syndrome (TS), chronic TD, and provisional TD, are characterised by the onset of tics, typically emerging around the age of 6 years and before the age of 18 [1]. Tics are repetitive movements or sounds that often present as jerking, grimacing, and coughing, and less frequently, coprolalia (explicit language) or copropraxia (offensive gestures) [2]. A recent National Institute for Health and Care Excellence (NICE) survey revealed that people waited between 6 months and 2 years to receive a diagnosis of either TS or chronic TD [3]. For adults with tics, these challenges can be further compounded due to the diagnostic criteria for TS requiring tics to be present before the age of 18 [4]. Therefore, both clinicians and patients must be confident that tics existed in childhood before a diagnosis can be made.

In the UK, healthcare support for tics begins with the general practitioner (GP) who, in most cases, will refer children and young people (CYP) to paediatric services or child and adolescent mental health services (CAMHS), while adults may be referred to neurology [5]. However, many patients report difficulties initiating a referral, with barriers including the GPs believing there to be no onward care available [5]. Even once referred, patients continue to report long waits to being seen by specialist services [6].

Doctors internationally reflect on receiving very little education on tics during their medical training, yet GPs report that they do not often see people with tics in their clinic [7]. However, with an estimated 300,000 people in the UK living with tics [8], it does raise the question whether a lack of knowledge of tics has contributed to an under-recognition amongst medical professionals. Furthermore, there are currently very limited NICE guidelines to aid GPs on how to assess tics or provide support for people with tics [9]. Alternatively, GPs are encouraged to refer to the European clinical guidelines for Tourette syndrome [10–12], which while comprehensive, does not always directly translate to healthcare practices in the UK. Consequently, with limited services and unclear pathways for tics nationally [13], offering and accessing treatment can be distressing, frustrating, and confusing for all parties [14]. A recent systematic review [7], highlighted the main issues in healthcare for tics as: lack of confidence in understanding and managing tics; presence of misconceptions and stigmas; inconsistent communication by healthcare professionals (HCP).

There is a clear need to strengthen GPs’ knowledge and awareness of tics to improve recognition and identification of tics, facilitate appropriate referrals, and provide longer term support to people living with tics. However, there is a lack of training currently offered to GPs on this topic. GPs in the UK are expected to accrue 50 credits, roughly equivalent to 50 h of continued professional development (CPD) each year [15]. Previous research in the UK demonstrated that 87% of GPs preferred to use online educational resources for CPD purposes [16]. There is also evidence to show that online learning resources are effective at improving GPs’ knowledge and confidence of neurodevelopmental disorders, such as attention-deficit/hyperactivity disorder (ADHD) [17]. Therefore, access to a well-informed online training resource could support knowledge development without overburdening GPs.

From the perspectives of both recipient and deliverer, this qualitative study aimed to explore the many aspects involved in primary care for tics, including healthcare support, referrals, and the overall patient experience. Understanding the needs of professionals and service users will inform the development of a future online intervention aimed at improving GPs' knowledge of tics.

## Methods

### Study design

Online semi-structured interviews were conducted in late 2024 and early 2025 by the lead researcher (AP). Ethical approval was obtained from the University of Nottingham (UoN) Mental Health and Clinical Neurosciences Ethics Sub-Committee in August 2024. The COnsilidated criteria for REporting Qualitative research (COREQ) checklist was followed for this study (see Additional File 1).

### Participants

The target sample was 20 participants across four groups: adults with tics; parents of CYP with tics; GPs; and other HCPs in secondary care. Eligible GPs had previously worked with people presenting with tics. Eligible HCPs had experience working with people with tics, and an understanding of primary care for tics either through working with GPs directly or through patient-reported experiences. For adults living with tics, and parents of CYP with tics, it was not necessary for them to have a TD diagnosis, so long as current investigations were indicative of a primary TD, not functional TD. Functional tics are sudden, involuntary movements or sounds that resemble TS tics but are often triggered by stress, anxiety, or underlying psychological factors [18]. They are a symptom of Functional Neurological Disorder (FND) and differ from typical, organic tics, as they often have a rapid onset, are more complex, and often occur in young adults rather than young children [18]. Other key differences of functional tics include the premonitory urge and the ability to suppress tics being more uncommon [18]. The diagnosis or assessment of a primary TD was determined through participant self-report. The criterion for only those living with primary tics being eligible was clearly highlighted to prospective participants in both the expression of interest form and participant information sheet (PIS). Recruitment through a charity and national steering group for primary TDs helped ensure participant eligibility.

Two adults with tics and one parent initially confirmed interest in the interview but did not respond to contact attempts. The lead researcher did not have any personal relationship to any of the people interviewed. Participants were aware that interviews were being conducted in fulfilment of a PhD thesis supporting the development of an online learning resource for tics, explained in the PIS.

### Data collection and analysis

A purposive sampling technique was employed, using an advert circulated to members of the National Tic Steering Group at the UoN and posted on the Tourettes Action website. Additional snowball sampling was used in the professional groups (GPs and secondary HCPs) to recruit other professionals using the same advert. With a focus on information power over saturation [19], the target sample size of 20 was deemed appropriate to explore perspectives from each of the highly specific groups, in an equally specific aspect of primary care.

The advert featured a link to an expression of interest form, where people could provide consent for the lead researcher to provide them with additional information about the study via email. Group-appropriate PIS and consent forms were sent to all who expressed interest. Following consent, all participants were sent a group-specific pre-interview questionnaire with clinical and demographic questions. Interview topic guides were also developed specifically for each group (see Additional Files 2–5).

All interviews were hosted on and audio-recorded using Microsoft Teams and transcribed using Microsoft Teams built-in transcription feature before the lead researcher (AP) reviewed for transcription errors and removed identifiable data. The audio recording failed for one participant, and while a transcript was still generated using MS Teams, the data was not included in the analysis as it was felt that without a recording the interview transcript could not be confidently reviewed for errors. Interview duration ranged between 24 min and 1 h 48 min. Each interview was only attended by the lead researcher and one participant, with no repeat interviews taking place. For quality assurance, all quotes used were verified against the recordings at the time of the manuscript preparation.

This study aimed to better understand the topic of healthcare for tics in England, not contribute or challenge a proposed theory, and therefore inductive thematic analysis was conducted. Following Braun and Clarke’s [20] six stage process, a period of data familiarisation took place during which initial thoughts were noted, before identifying preliminary codes.

Initial coding was conducted line-by-line to capture notable features of the data, generating a set of preliminary codes grounded in participant accounts. These codes were then compared, refined, and grouped into themes through regular discussions within the research team. A coding framework was developed in Microsoft Excel to map the relationships between codes, subthemes, and overarching themes, and was iteratively revised as analysis progressed. The coding tree can be found in Additional File 6. This process aimed to ensure that themes accurately reflected patterns across the dataset. A portion of the transcripts were reviewed by second coders, CLH and BF, to establish quality and agreement.

### Reflexivity

The lead author is a cisgendered White British male who has previous clinical experience working in psychology and occupational therapy teams before transitioning to child and adolescent mental health research. They have frequently collaborated with other researchers and clinicians in neurodevelopmental, neurological, and child and adolescent mental health research where the challenges of people with living with tics is well understood. It is important to consider that the lead author who conducted the interviews has been researching tics more recently as part of their PhD and acknowledges the expertise of interviewees is far greater than their own. This supported an interpretivist approach to data analysis, placing greater value on the insight of participants as the experts of their own experiences. However, it is acknowledged that researcher expertise may affect the information power of the data presented. The lead author had been previously trained to conduct interviews as part of their involvement in other mental health research projects, with additional support by supervisors/co-authors with experience in qualitative methods.

## Results

Six GPs, four HCPs in secondary care, five parents, and five adults with tics (*N* = 20) were interviewed on their experiences and perspectives of healthcare for tics in primary care. Participant demographics are illustrated in Table 1, and parent-reported children and young people (CYP) demographic data is displayed in Table 2. A flowchart of participant recruitment can be found in Additional File 7.

**Table 1:** Participant demographic information

Participant (*N* = 20)	GP	HCP	Parents	Adult with Tics
N	6	4	5	5
Participant Age				
26–35	1	-	-	1
36–45	2	-	3	2
46–55	3	2	2	1
56–65	-	2	-	1
Participant Gender				
Female	3	2	4	2
Male	3	2	1	3
Participant Ethnicity				
Asian	2	1	1	1
White	4	3	4	4

‘-‘ denotes that no participants were recruited into that age bracket for each given group

**Table 2:** CYP demographic information, as reported by parent participants

CYP (*n* = 5)	CYP demographic (reported by Parents)
Age	6–18
Gender	
Female	2
Male	3
Ethnicity	
Asian	1
White	4

CYP demographic information was reported by Parent participants in the pre-interview questionnaire. No CYP were recruited for interview

Of the patient groups, all adults and CYPs (as reported by their parent) had either received a TD diagnosis or were currently under investigation. It was self-reported by adults with tics to have waited between 1 and 10 years for a diagnosis to be confirmed. Parents reported waiting between 5 months and 4 years for their young person’s diagnosis to be confirmed through the National Health Service (NHS), while one young person diagnosed within 5 months had accessed private healthcare. When tics first emerged, the time taken to seek first-line support was between 4 months and 4 years, when tics first became more frequent, or in adulthood despite having them in childhood. Parents sought support after the first tic emerged, in the first few weeks, or up to a year later.

For the clinical groups, GPs had been in practice between 6 and 22 years, with most seeing between 1 and 3 people with tics annually, and one seeing more than 10. Most GPs report little to no training during their medical education, with just one GP spending a day in a tic clinic as part of their clinical placement. Most GPs developed their knowledge of tics through CPD opportunities. HCPs in secondary care all worked with people up to the age of 18 with most seeing more than 10 people annually with tics and have completed CPD opportunities to develop tic knowledge. A summary of the three themes that were generated from the data can be found in Additional File 8.

### Factors impacting GP knowledge and education of tics

#### Limited commissioned training opportunities and guidelines

All GPs reported receiving very little education on tics disorders in their medical training. Some GPs could recall reading a few lines in a textbook, but nothing formally as part of the curriculum.

*“It’s non-existent. All right*,* I might have had some tiny*,* you know*,* a line of it or something in my years ago medical school. But it’s*,* yeah*,* definitely not something that has featured in my initial training or many of my updates that I’ve had over the years.*” – Participant 21, GP.

However, beyond taught material, some GPs recall encountering tics during paediatric and mental health placements. One GP recalls learning about the diagnostic criteria for TS on their paediatric placement, but not how they could support families. Others noted that not all GPs complete a placement in paediatrics, therefore limiting their exposure to tics in clinical practice.

*“I think as a GP in my GP training*,* I’ve not received any specific training on tics. It’s just purely that I’ve done a placement in mental health and some GP trainees*,* I guess most GPs do get mental health as a as a placement block. But not everyone*,* to be honest. And a lot of their experience is probably going to be in the acute hospitals rather than in the outpatient clinics.”* – Participant 20, GP.

One GP noted that from their experience, new trainees were not finishing their medical training with the necessary communication skills.

*“The clinician needs to be confident*,* competent and comfortable talking about tics and have excellent advanced primary care consultation skills. And I’m sorry to say that a lot of the trainees in my area have a high failure rate and many of them are not leaving the training scheme with such good quality consultation skills as I would like them to have in this context of tics.”* – Participant 14, GP.

This sentiment was recognised by other groups, who also recall feeling as though GPs lacked knowledge about tics. Additionally, it was common for adults with tics and parents to see locum GPs who were thought to be less familiar with tics than salaried GPs, making continuity of care more challenging.

*“There’s a few very long-established GPs who are very sensible through experience*,* but the difficulty is that a lot of primary care is now delivered by locum GPs who are not as familiar with any disorders*,* let alone tics.”* – Participant 17, HCP in secondary care.

#### Everything but a tic disorder

Adults with tics, parents, and secondary HCPs varied in their experience when recalling GP knowledge of tics, but often respondents reported that their GP was unfamiliar with the condition or mischaracterised tics with other conditions, some possibly life-threatening, including epilepsy or brain tumours.

*“Well before my diagnosis could be fully confirmed*,* I had to go through a process of elimination of what it is not*,* “let’s rule out it’s not this”. Initially the first thing they thought was possibly I had a brain tumour to explain the movements and different things*,* so it was just a very*,* very anxious time and uncertain time.”* – Participant 6, Adult with Tics.

While GPs expressed the importance of ruling out serious health concerns, this further increased patient anxiety and occasionally resulted in inappropriate medications being prescribed, including nasal sprays for allergies. Adults with tics and parents reported that each presentation to the GP was considered in isolation, instead of considering previous contact history to build a greater picture of their experience.

*“Because the amount of families that have been referred to ophthalmology because of eye tics or they’ve been referred to allergy clinics because of cough*,* a consistent cough. Or you know*,* they’ve had all their allergy tests done for lactose intolerance and whatever. The assumption is not that it’s a tic and 20% of kids have got tics*,* you know*,* at some point in their life.”* – Participant 12, HCP in secondary care.

#### Individualised barriers and facilitators of tic knowledge

All groups discussed factors they felt made identifying tics and appropriately supporting people easier or more challenging. The most common factors included the number of people encountered with tics, personal experience of tics, self or family member, and general interest in tics and TDs.

*“Initially*,* my doctor at the time was very inquisitive. He was curious. He was very pro-wanting to know more and I found that*,* you know*,* the treatment has been hit and miss with other GPs basically based on the fact of their interest in it. You know if they’re like… if they want to do more about it*,* they’ll educate themselves a lot more about it.”* – Participant 6, Adult with Tics.

GPs with more experience and interest in tics became more specialist within their practice. However, this may have been at the detriment to colleagues, as they become less likely to see new tic cases or be less incentivised to develop their knowledge.

*“Massively so. So I mean most GP surgeries will have GPs with a special interest if typically you’ll have someone who’s*,* you know*,* really hot on dermatology*,* and so they’ll tend to see the skin lesions and things like that. So and then everyone else*,* and I’ve asked them for advice*,* I guess I’m the equivalent for tics and Tourettes. I’d hope that they’re all much better at it now*,* because I’ve done quite a few education sessions for them. But the danger of having some in your practice is that everyone else de-skills*,* but hopefully they’re all aware of Tourettes Action and that but they do just now send them to me so.”* – Participant 18, GP.

#### Balancing responsibility and pressures as a GP

GPs acknowledge that they are the gatekeepers for people wanting to access additional support for tics in secondary care. However, GPs also expressed concern as they are expected to know a lot about many conditions, experiencing pressure from various groups and charities to ensure their condition is represented and understood. Regarding tics, GP recognition of these limitations varied with some believing they could be doing more, including diagnosing TDs in primary care and reducing the pressure of waiting lists in secondary care.

*“I think it’s entirely possible for Tourettes to be managed the way we manage many*,* many*,* many conditions*,* as GPs. With common stuff with children*,* I think asthma is a really good example*,* is I can see a child. I can diagnose that child. I can treat that child without involving any secondary care conditions.”* – Participant 18, GP.

As gatekeepers and professionals, adults with tics and parents discussed the trust they placed in GPs to appropriately assess their symptoms and make suitable recommendations, demonstrating the reliance of primary care. However, others felt they had to educate themselves and present this to their GP to progress their care.

*“I was diagnosed with touretteism which I don’t even know as a word. But I think the main thing and the pattern all the way through this is that I’ve been the one who’s had to go back with the information*,* and it’s been up to me to research and educate myself about the condition. Otherwise*,* I would have been in that vicious circle of just taking antipsychotics just to suppress and hold my tics in.”* – Participant 6, Adult with Tics.

### Inconsistent availability of support for tics

#### Challenges accessing support services

GPs and HCPs discussed how acquiring a TD diagnosis was often necessary to access any additional support. However, all groups recalled common challenges in secondary care for tics, including the limited provision of services across the country, with some believing there to be better support in south of the country compared to the north. Even more specifically, it was understood that while some regions offered children services, others only had adult services, both raising doubt around the continuity of care and the ability to support adults undiagnosed as children.

*“the GPs don’t actually know who to refer to because the paediatricians won’t see*,* the neurologist won’t see and CAMHS won’t see*,* so they don’t actually have anybody to refer to.”* – Participant 17, HCP in secondary care.

Clinicians in both primary and secondary care felt disillusioned by the lack of support available to those with tics, while adults with tics and parents report the implementation of inappropriate treatment and failure to anticipate future needs of children when moving from primary to secondary school.

*“I think that that causes a barrier in itself because GPs*,* like us*,* are frustrated*,* they’re feeling disillusioned. And so there is a sort of*,* you know*,* what’s the point in sending off a referral because it’s not going to make any difference”* – Participant 17, HCP in secondary care.

However, it was suggested that a lack of specialists was not exclusive to tics, alluding to wider concerns around the supply and demand of services in the healthcare sector. Of those who accessed private healthcare for tics, adults with tics and parents report shorter waiting times and improved accessibility, but little on the quality of care they received, further implying a need for more specialists and better access to treatment.

*“But the main problem is actually there’s just not enough doctors and specialists out there to cope with the volume of paediatric referrals altogether*,* let alone absorbing the tics on top of it. So the main thing is actually we just don’t have the resources.”* – Participant 17, HCP in secondary care.

#### Referral complexities and challenges

Beside a lack of dedicated services to support tics, several factors were identified which made completing a referral more difficult, even in areas that appeared to have specialist services. The most endorsed challenge was the lack of well-defined processes to access referral services. It was also reported by parents that there needed to be a clear mental health component for a referral to be made/accepted. However, while HCPs suggest this was to manage service capacity, it also meant that tic referrals were not appropriately allocated to a service equipped to manage tics.

*“But neurodevelopmental mental health conditions aren’t even owned by mental health services because they see it as kind of like physical that “yeah*,* that’s paediatrics. Then we’ll do self-harm and depression and anorexia and stuff like that”. So this kind of middle ground that no one really knows what to do with it.”* – Participant 12, HCP in secondary care.

Professionals in secondary care reported that occasionally referrals did not contain sufficient information about tics. Interestingly, interviewed GPs recognised the benefit of video evidence and letters from school and the importance of including patients in writing the referral, however, parents and adults with tics report not being asked to provide any such evidence. Since adults with tics and families were not often included in the correspondence between primary and secondary care, it was then the responsibility of the GP to relay the news of rejected referrals to families, further exacerbating their confusion and anxiety and potentially rupturing the therapeutic relationship.

*“And when referrals are rejected*,* the patient or carer is not normally copied in this*,* thus leaving me with the liability… They talk about rejected referrals*,* which I think is not therapeutic for patients. And I think there’s a lot of research to be done about the language we use in our consultations in primary care because the consultation skills*,* the language*,* the verbal and nonverbal communication are key to whether our interactions are therapeutic.”* – Participant 14, GP.

### Experiences in primary care for tics

#### Stigma, dismissal, and denial of tics

Many interviewees reported that common misconceptions included the belief that tics were voluntary or indicative of attention-seeking behaviour and overestimating the frequency of coprolalia as a presenting symptom. Stigmatising misconceptions about tics were held by adults presented with a TD diagnosis, families, primary and secondary HCP, and schools. This indicates a broader misunderstanding of tics and TDs which exacerbates misconceptions and harmful beliefs. Despite groups recalling recent positive celebrity awareness campaigns, the over-representation of coprolalia in the media continues to be most noteworthy, instilling stigma and making it more difficult for some to accept a TD diagnosis.

*“If you say Tourette’s syndrome*,* it is usually the kind of very rare cases which are portrayed in media and sometimes families are quite worried when the diagnosis comes in. And actually it is not the case. It’s a very rare situation when there is coprolalia where they see where they say very obscene words and people kind of think that that a Tourettes diagnosis it’s*,* it’s what they saw on the TV. And then we have to kind of dial it back a bit and tell them about what is the criteria and how that is quite rare to have such kind of presentation”* – Participant 15, HCP in secondary care.

GPs identified that due to service demand, support was often primarily offered to those with more severe tics, causing adults with tics and parents to feel dismissed when their/their child’s symptoms were played down. Consequently, HCPs recognised the belief that symptoms had to get worse before being taken seriously or were deserving of more support. In a desperate attempt to be seen, one interviewee exacerbated their tics and developed an abnormally fixed posture, causing additional physical pain.

*“the biggest health misconception is that it’s severity and I have to wait until it’s a severe problem to refer or I can only refer that. I can only refer something that’s a problem*,* you know. It has how you judge impairment and how you judge severity.”* – Participant 12, HCP in secondary care.

#### Understanding the individual impact of tics

Adults with tics and parents recall the wider impact tics can have when socialising with peers, pain caused by repetitive movements, fatigue levels, and academic engagement. However, while GPs report the importance of understanding impact and building a clinical history, patient groups and HCPs recall a lack of consideration beyond visible symptoms.

*“I think there is a gap in understanding of how significant tics can be in terms of pain*,* mental health difficulties and the impact on daily life. I think there’s tends to be a “well*,* there’s little movement or a little sound. What’s the concern about it?”.”* – Participant 17, HCP in secondary care.

*“A lot of the stuff that’s written about meetings is*,* “oh*,* they’re doing well”*,* “they’re fine”*,* “they’re OK”*,* and “they’re dealing well with their tics”. And I often think they just got*,* they’ve really not understood*,* you know*,* in that 15–20 minute or 10-minute GP appointment. It’s not really understood what is going on for a family at all”* – Participant 8, Parent.

#### Communication and educating patients about tics

Many adults with tics and parents recall being offered little information about tics when approaching their GP, with some only being signposted to charity websites when they were hoping for NHS advice or pain-relieving exercise plans. GPs also recalled offering externally available resources for tics online, including charity leaflets, social media, and support groups. This brings into question whether a lack of signposting is a result of few resources, or a characteristic of inadequate patient communication and consultation style. Most adults with tics and parents reported little reassurance or empathy displayed by their GPs. However, GPs reflected on their approach to patient communication, balancing pragmatism and false hope given the lack of service availability, guidelines, and primary care knowledge. One GP did reflect on the variation in the level of consultation and communication skills amongst GPs, especially in the context of tics. This suggests there may be shortcomings in the communication around tics when responding to patient concern, and the availability and awareness of appropriate signposting information for families.

*“I think there’s a really fine balance between reassuring people and people feeling not heard and dismissed. And I know in the Tourettes community*,* a lot of what is felt is that they’re being dismissed. And I can see there’s a big disparity.”* – Participant 18, GP.

*“Of course*,* for the patient*,* it should feel holistic and sympathetic*,* and I’ll make sure I say things like “that’s very difficult for you”*,* “That must be very hard for you”*,* “How does that make you feel?”*,* “It must be embarrassing for you”. I would be doing all of those things*,* of course.”* – Participant 14, GP.

## Discussion

The thematic analysis of primary care experiences across different groups generated three interrelated themes covering the identification of tics, inconsistent access to specialist/secondary services, and a range of generalised and individualised factors shaping care experiences. The qualitative methodology encouraged a nuanced exploration of the perspectives of people living with tics and those involved in their care.

*Factors Impacting GPs’ Knowledge and Education of Tics* included investigating differential diagnoses, few training opportunities, individual clinical interests and presentations, and the balance between GPs’ responsibility and patient expectations. Patient groups and secondary care HCPs felt GPs did not always consider tics in consultations, meaning various other health conditions were investigated first, including brain tumours and epilepsy, causing additional unnecessary worry for patients. GPs reported limited medical training on tics, with pockets of knowledge originating from personal experience, interest, and the number of presentations in clinic. Rising demands on GPs mean that designating GP-specialists may relieve workload pressures, but risks reducing expertise among colleagues in managing specific conditions, such as tics. However, some GPs felt they could be doing more to alleviate demand further along the pathway in secondary care, including diagnosing TDs which one GP was already doing. Previous research has shown that patients viewed HCPs as having insufficient knowledge of tics. This was demonstrated through insufficient clinical investigations [5] and limited support or signposting [21], even in instances where GPs demonstrated some knowledge of tics [7, 22]. Negative experiences appear to stem from a lack of confidence due to infrequent case presentations and limited training opportunities. Expectedly, time pressure and fear of mistakes were paramount to experiences of stress in previous research interviewing GPs about workplace stressors [23]. GPs’ views varied in their sense of responsibility for managing tic cases, with some believing secondary services or the patients themselves should take more responsibility. These findings suggest that improved access to manageable training would enhance GPs’ knowledge and confidence in managing tics in primary care.

*Inconsistent Availability of Support for Tics Across the Country* was discussed by all groups, including the geographical sparsity of services. It was often unclear whether Paediatric Neurology or Child and Adolescent Mental Health Services (CAMHS) was most appropriate for CYP. Additionally, CAMHS required a clear mental health component associated with tics before a referral could be accepted, potentially limiting tic-specific support. This was confirmed by Rattu, Hall [13] who reported very few service pathways for CYP with tics in England. Neurodevelopmental services did not always accept cases of tics unless ADHD and autism spectrum disorder (ASD) were the primary reason for referring [13]. In the present study, some GPs were aware of tic specialist services but were unable to refer directly. Instead, they referred to Paediatrics before being triaged to the most appropriate service and further delaying access to support.

HCPs in secondary care reported that some referrals did not include sufficient information to accurately assess need, resulting in the referral being declined. These inconsistencies are likely to exacerbate the already lengthy waiting times for secondary care within England [5, 24]. GPs face challenges in gaining all relevant information before making a referral given the shift to short, often remote appointments in primary care settings. Standardised referral processes are likely to enhance the quality of referral information [25] and would further benefit from direct liaison with schools, workplaces, and families to gain a broad picture of daily impact. While GPs endorsed the usefulness of supporting evidence, including videos, most adults with tics and parents report not being asked to provide any evidence. Improving GPs’ knowledge of tics, clarifying the most appropriate locally available pathway, and encouraging evidence from families could facilitate accurate referrals and reduce confusion.

Effective non-pharmaceutical treatments exist, including Comprehensive Behavioural Intervention for Tics (CBIT) and habit reversal therapy (HRT) [26, 27]. However, these are often unavailable due to a lack of expertise and funding [5, 13, 28]. Systemic resourcing improvements for services for people with tics would result in better access to treatments that when paired with refined referral processes may greatly improve patient experiences.

The theme of *Experiences of Primary Care for Tics* confirmed that stigma and misconceptions remain an issue for people living with tics. All groups discussed how many people still assume tics are voluntary and that coprolalia is common, and the role media has on experiences. For example, media representations were suggested to occasionally improve public perceptions of TD. However, the overrepresentation of coprolalia made it more difficult for people who did not experience coprolalia to accept a TD diagnosis. Recent research has found that media portrayal of TS continues to overstate coprolalia as a primary symptom, exacerbating stigmatising beliefs and misconceptions [29], including in primary care [7, 30, 31].

Media representations occasionally brought attention to more severe cases of tics. However, some adults with tics felt their symptoms were not severe enough to warrant support. Meanwhile, parents were encouraged to wait until tics caused pain or distress before the GP would agree to refer their child. This misconception was identified by an HCP working in a tic service. They instead suggested that referrals should consider the many facets of impact in addition to visible symptoms and pain, including mental health and functional ability of living with tics. However, since tics can be transient, GPs may have been hesitant to refer before confirming the onset was chronic in nature. This is especially likely when existing pressures are prolonging wait times into secondary services. However, this could highlight a breakdown in communication whereby clinical decisions are not discussed with people presenting with tics and their families. Either way, suitable education could dispel problematic beliefs and communication with families, improving the therapeutic relationship and facilitating more appropriate management.

The results of this study reflect the conclusions of a systematic review exploring healthcare experiences for TDs internationally, including the UK, US, Canada, Saudi Arabia, Uganda, and Australia [7] (see summary of included studies in Additional File 9). For example, HCPs, including those in primary care, consistently reported few training opportunities [30]. This impacted their confidence in recognising and appropriately managing tic presentations. However, many were keen to further their understanding and offered content recommendations for resources [32, 33]. People recalled their tics symptoms often being misinterpreted [32, 34], feeling misunderstood, and communication that lacked clarity [22, 34]. Collectively, these all exacerbated the impact of living with tics and contributed to negative experiences with health services, which is not exclusive to the UK.

The implications of this study reaffirm that tics remains a largely unfamiliar and misunderstood condition, not just for GPs but for HCPs more generally. Content covering tics does not feature in the core learning curriculum for medical students, nor has it been frequently completed as part of ongoing CPD. Consequently, the patient experience for TDs in primary care continues on a negative trend. A lack of standardised education does not equip HCPs, including GPs, with the necessary clinical skills to thoroughly investigate tics. An early value assessment (EVA) of digital therapies including the Online Remote Behavioural Intervention for Tics (ORBIT) [6, 28] proves promising. However, access to comprehensive NICE guidelines are required to streamline investigation and delivery of care for tics. While it is important to rule out other serious conditions, inadequate education engenders anxiety and confusion through delayed diagnosis and access to support. Improved education is likely to encourage more robust investigation of the impact of tics and the submission of more information-rich referrals. This will ensure that people are allocated to the most appropriate service with a clear understanding that tics are the primary concern for the individual. Resourcing concerns continue within the NHS as service demand grows [35, 36]. Therefore, improving education and awareness of tics will stimulate the generation of formalised guidelines and the allocation of resources to implement a dedicated healthcare pathway in England.

The findings of this paper will inform the development of a GP-tailored online learning resource that considers the additional needs identified by peoples accessing primary care for tics and secondary care HCPs communicating with primary care. The resource intends to offer information on a range of tic presentations, patient communication, impact awareness (pain, sociability, academia, hidden tics, and mental health), common misconceptions and stigma, and useful signposting information and resources. It is important for the resource to be time-manageable and focusing on content specific to the role of a GP in managing tics. Further research should explore the disparity of healthcare for TDs in the underserved LGBTQIA + and ethnic minority communities that already experience serious health inequality and discrimination. Collectively, this will build evidence necessitating the need for robust NICE guidance and the development of dedicated services with a clear referral pathway.

### Limitations

Participants were recruited from England, and while geographically diverse, we were unable to recruit any participants from Wales, Scotland, or Northern Ireland. While there are few tic specialist services within England [13], none exist in Wales and Scotland, potentially leading to further challenges and uncertainty for patients and families [37]. Additionally, a 2023 study found that a majority of GPs in Wales, Scotland, and England were unsatisfied with their work-life balance [38]. Consequently, this suggests that patient and professional experiences may be similar across the UK, further substantiating the need to better support GPs.

Professionals were recruited through the UoN TD networks and further snowball sampling. From the interviews, it was apparent that some GPs had more personal interest in tics, which is unlikely to reflect the general population of GPs. Interestingly, many of the needs raised by the adults with tics and parent groups were also highlighted by the GPs who were more knowledgeable about tics. Therefore, while the sample may not be representative of the wider GP population, it does offer some insight into how primary care could benefit from improved knowledge of tics, including the intention to understand the deeper impact of tics. Although the sample of each group was small, this is in line with previous recommendations [20, 39], including TD research [40]. Parental viewpoints were relied upon since CYPs were not interviewed. While parents may often lead discussions in clinical settings, interviews with CYPs may have generated interesting data about self-advocating and the parent-child-professional dynamic in consultations.

Unfortunately, a restricted study timeframe meant some practices that would have benefitted data-credibility were unable to be completed. These included gathering participant comments and corrections on interview transcripts, approaching participants for feedback on findings, and piloting the interview topic guides.

## Conclusion

This paper builds upon a limited body of literature in patient and professional experiences of tics in primary care. Some GPs are keen to develop their understanding following limited opportunities during medical school. A sparce availability of resources results in negative patient experiences when considering their GP’s ability to support their care needs. The development of an online learning resource for GPs could facilitate improved knowledge of tics and the understanding of impact beyond the immediate symptoms, therefore supporting appropriate, informed and timely referrals to secondary care. Across all care sectors, current support for individuals with tics remains insufficient. However, ongoing efforts to educate those in professional care positions, developing a dedicated referral pathway, and integrating evidence-based behavioural therapies will improve clinical competency and patient outcomes.

## Supplementary Information

Supplementary Material 1.

Supplementary Material 2.

Supplementary Material 3.

Supplementary Material 4.

Supplementary Material 5.

Supplementary Material 6.

Supplementary Material 7.

Supplementary Material 8.

Supplementary Material 9.

## Data Availability

The data generated during the current study are available from the corresponding author upon request.

## Abbreviations

ADHD: Attention Deficit/Hyperactivity Disorder
ASD: Autism Spectrum Disorder
CAMHS: Child and Adolescent Mental Health Services
CBIT: Comprehensive Behavioural Intervention for Tics
COREQ: COnsolidated criteria for REporting Qualitative research
CPD: Continued professional development
CYP: Children and young people
EVA: Early value assessment
FND: Functional Neurological Disorder
GP: General practitioner
GP-TiCS: Growing Proficiency in Tic Disorder Care and Skills
HCP: Healthcare professional
HRT: Habit reversal therapy
LGBTQIA+: Lesbian, gay, bisexual, transexual, queer or questioning, intersex, asexual, and more
NHS: National Health Service (UK)
NICE: National Institute for Health and Care Excellence
NIHR: National Institute for Health and Care Research
ORBIT: Online Remote Behavioural Treatment for Tics
PIS: Participant information sheet
TD: Tic disorder
TS: Tourette syndrome
UoN: University of Nottingham
