# Supplementary material for: Growing Proficiency in Tic Disorder Care and Skills (GP-TiCS): a thematic analysis of patient and professional group experiences in primary care for primary tic disorders
Source: BMC Prim Care. 2026 Jul 30;27:388. doi: 10.1186/s12875-026-03496-y (PMC13711887; doi:10.1186/s12875-026-03496-y)
Supplement: Supplementary file 2 [file 12875_2026_3496_MOESM2_ESM.pdf]

Additional File 2

Topic guide for Adults with Tics participant group

Need for Education

1. Can you tell me about your experience with your GP for your tics?
    a. How well did you feel your GP understood tics?
2. Can you describe your initial appointment with your GP when presenting with tics?
3. What did your GP tell us as to why you were experiencing tics?
4. Did your doctor offer any support or education on tics?
    a. If so, what did they offer and was it useful?
5. How would you describe your GPs confidence when discussing tics with them?

Communication of Tics

1. How did your GP communicate to you about tics?
    a. Did you feel included in any decisions made, and why?
2. Did your GP offer any reassurance, and if so, what did they say?
    a. How would you describe their empathy and attentiveness?

Misconceptions and Stigma

1. What were your GP's views or attitudes towards tics or tic disorders?
    a. Can you describe how your GP initially responded to your concerns?
    b. How did your experience compare in secondary care?

Referral Process

1. Did your GP refer you to a specialist doctor or service (e.g., tic specialists, neurologist, psychiatrist, CAMHS)?
    a. How did you find the referral process for tic with your GP?
    b. Did they keep you updated on any progress, and how?
    c. How long did you have to wait for a referral to be completed?
2. Did you feel included by your GP in any decision making during the referral process, and why?
3. Did your GP ask for any additional information before a secondary care referral could be made, e.g., familial report or clinical observation?
4. Can you describe the communication between your GP and secondary care services about your tics, e.g., information provided, language used?

Impact

1. How have your experiences of healthcare for tics impacted your quality of life in either a positive or negative way?
2. Is there any information you wish you were initially told by your GP?
    a. Was there any information about tics you wish your GP knew more about?
3. Were you diagnosed with a tic disorder in adulthood?
    a. How do you feel your experience may have been different, if at all, because you were an adult presenting with tics?
        i. Do you think your concerns were taken more or less seriously than a child presenting with tics?
    b. Has your experience with GPs regarding tics changed over time, and how?

Education and Training

1. What is your opinion on GPs receiving additional training on tics and tic disorders?
    a. Is there any content you think would be important for them to know more about?

Any other information

1. Is there any other information you would like to mention based on the questions you have been asked today?
