# Supplementary material for: Growing Proficiency in Tic Disorder Care and Skills (GP-TiCS): a thematic analysis of patient and professional group experiences in primary care for primary tic disorders
Source: BMC Prim Care. 2026 Jul 30;27:388. doi: 10.1186/s12875-026-03496-y (PMC13711887; doi:10.1186/s12875-026-03496-y)
Supplement: Supplementary file 4 [file 12875_2026_3496_MOESM4_ESM.pdf]

Additional File 4

Topic guide for General Practitioner participant group

Need for Education

1. What are your beliefs and understanding of tic disorders, e.g., symptoms, diagnosis criteria, treatments?
    a. Do you consider there to be any gaps in your understanding, and if so, what are they?
2. How confident do you feel working with people with tics?
    a. What aspects do you feel most confident about?
    b. How would your confidence compare to working with people with ADHD or OCD?
3. Do you feel your understanding of tics compares to your colleagues, and how?
    a. Do you feel this compares to clinicians in secondary care you have had contact with, and how?
4. How would you rate your understanding of tics and tic disorders?

Communication with Family

1. What information do you think is important for families to know about tics?
    a. How do you involve family in any decision-making?
2. How important do you think it is for families to be involved in decision-making for tic support?

Stigma and Misconceptions

1. Have you heard or observed stigma and misconceptions related to tics, if any?
    a. If so, what were these?
2. Do you think a tic disorder diagnosis is important, and why?

Referral process

1. What is your understanding of the referral process for tics?
    a. Are you aware of tic specialists or services?
    b. At what point do you think a secondary care referral is necessary?
2. What information do you use to inform a referral for tics, and what are these?
    a. Examples - teacher reports, clinical observation, quality of life assessments.
3. How do you find the communication to be between primary and secondary care regarding a tic referral?
4. Roughly how long does it take for a referral request for tics to be completed?
5. How would you rate your understanding of the referral process/pathway for tics?
6. Can you describe any positive or negative aspects of the referral process?

Barriers and Facilitators

1. Can you describe your experience working with people with tics?
2. From your experience, can you think of any facilitators when supporting people with tics? If so, what are these?
    a. What could be done to promote the facilitators to occur more frequently?
3. Do you consider there to be any barriers/challenges when working with people with tics? If so, what are these?
    a. How would you attempt to overcome these challenges?
    b. Could anything have prevented these challenges?

Education and Training

1. How do you feel about the training you have received on tics and tic disorders?
    a. Can you describe the quality of training you received, considering positive and negative aspects?
2. What is your opinion on receiving additional training on tics and tic disorders?
3. How feasible would you find it to complete additional training?
    a. When would you do the training?
    b. Could it be completed as part of your CPD?
4. How would you go about completing additional training for tics
        i. Would a manager need to be informed?
5. What would be the most suitable format for additional training?
6. What format do you find most beneficial when completing online learning (e.g., videos, activities, text)?
    a. What is a manageable time commitment for completing the resource?

Additional Information

1. Is there anything else you would like to mention based on the questions you have been asked today?

Invitation to Workshop

1. Once an outline of the learning resource has been developed, would you be willing to attend a workshop to further discuss the content and format of this?
