# Supplementary material for: Growing Proficiency in Tic Disorder Care and Skills (GP-TiCS): a thematic analysis of patient and professional group experiences in primary care for primary tic disorders
Source: BMC Prim Care. 2026 Jul 30;27:388. doi: 10.1186/s12875-026-03496-y (PMC13711887; doi:10.1186/s12875-026-03496-y)
Supplement: Supplementary file 5 [file 12875_2026_3496_MOESM5_ESM.pdf]

Additional File 5

Topic guide for Healthcare Professional participant group

Need for Education

1. How well do you believe GPs understand tics and tic disorders, e.g., symptoms, diagnosis criteria, treatments?
    a. Have you known any GPs to hold certain beliefs about tics, and what were they?
    b. Do you consider there to be any gaps in GP understanding, and if so, what are they?
    c. Were there areas you felt GPs were more knowledgeable about than others?
2. How confident do you feel working with people with tics?
    a. How confident do you perceive GPs to be when working with people with tics?
3. Do you think the understanding of tics compares for clinicians in primary and secondary care?
4. How would you rate GP's understanding of tics and tic disorders?

Communication with Family

1. Do you have any thoughts on the communication between GPs and families about tics, and if so, what are these?
2. What information do you think is important for GPs to discuss with families about tics?

Stigma and Misconceptions

1. Are you aware of tic-related stigma and misconceptions, and what were these?
2. Have you heard or observed stigma and misconceptions related to tics from GPs, and what were these?
3. Do you think a tic disorder diagnosis is important, and why?

Referral process

1. What is your understanding of the referral process for tics?
    a. What is your opinion on the referral process?
2. Do you think the tic pathway is clearly understood by GPs?
    a. What information do you require from GPs to action a referral request?
3. How do you find the communication to be between primary and secondary care regarding a tic referral?
4. Roughly how long does it take for a referral request for tics to be completed?

Barriers and Facilitators

1. Can you describe your experience working with GPs on a referral for tics?

2. Do you consider there to be any facilitators when working with GPs on a referral for tics? If so, what are these?
    a. What could be done to promote the facilitators to occur more frequently?
3. Do you consider there to be any barriers/challenges when working with GPs on a referral for tics? If so, what are these?
    a. How could these challenges be overcome?
    b. Could anything have prevented these challenges?

Education and Training

1. What is your opinion on GPs receiving additional training on tics and tic disorders?
    a. What content do you think would be most important for them to know more about?

Additional Information

1. Is there anything else you would like to mention based on the questions you have been asked today?
