# Supplementary material for: Growing Proficiency in Tic Disorder Care and Skills (GP-TiCS): a thematic analysis of patient and professional group experiences in primary care for primary tic disorders
Source: BMC Prim Care. 2026 Jul 30;27:388. doi: 10.1186/s12875-026-03496-y (PMC13711887; doi:10.1186/s12875-026-03496-y)
Supplement: Supplementary file 6 [file 12875_2026_3496_MOESM6_ESM.pdf]

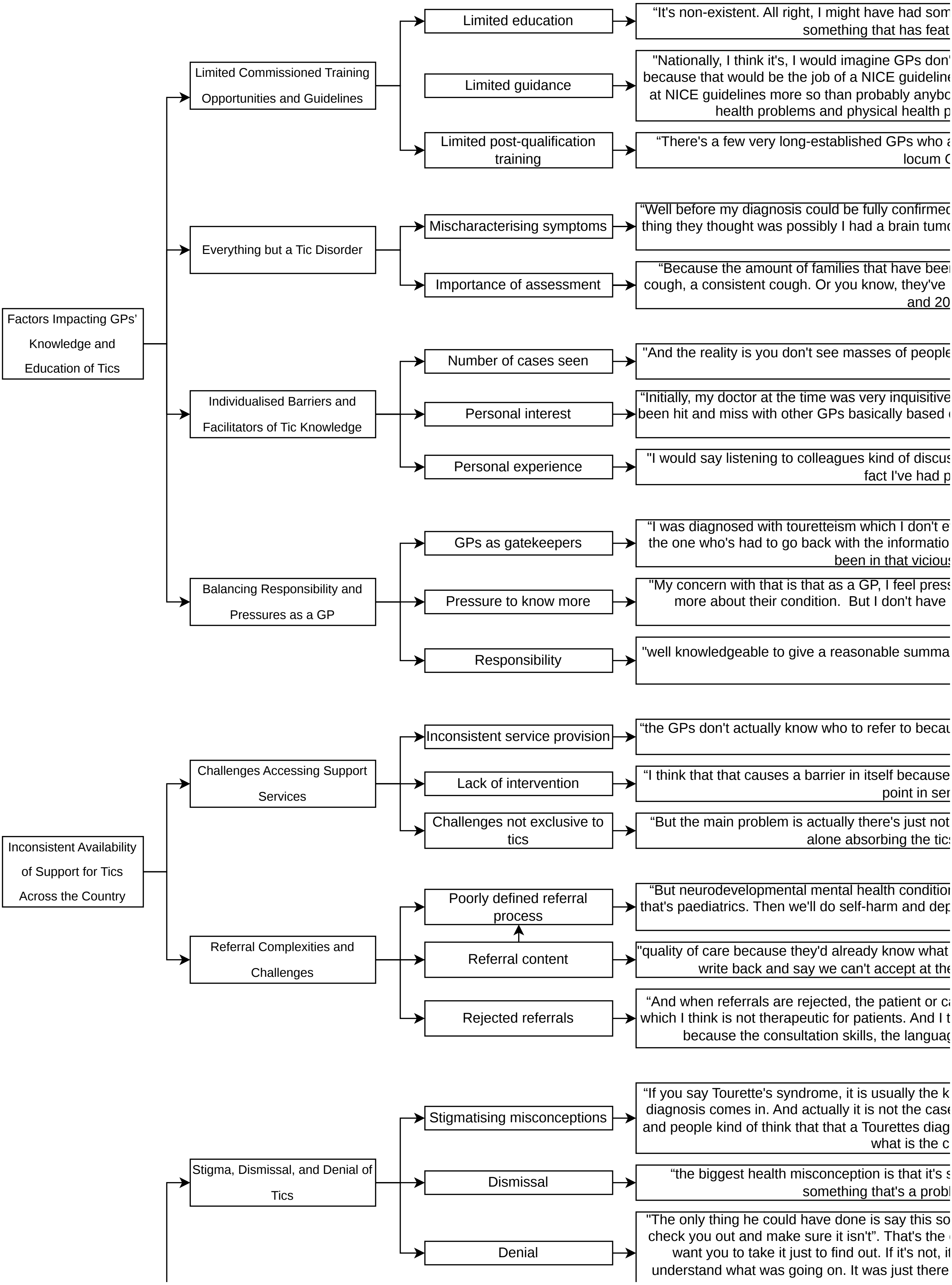
Factors Impacting GPs' Knowledge and Education of Tics
Limited Commissioned Training Opportunities and Guidelines
Limited education
"It's non-existent. All right, I might have had som
something that has feat
Limited guidance
"Nationally, I think it's, I would imagine GPs don'
because that would be the job of a NICE guideline
at NICE guidelines more so than probably anybo
health problems and physical health p
Limited post-qualification training
"There's a few very long-established GPs who a
locum G
Everything but a Tic Disorder
Mischaracterising symptoms
"Well before my diagnosis could be fully confirmed
thing they thought was possibly I had a brain tumo
Importance of assessment
"Because the amount of families that have been
cough, a consistent cough. Or you know, they've
and 20
Individualised Barriers and Facilitators of Tic Knowledge
Number of cases seen
"And the reality is you don't see masses of people
Personal interest
"Initially, my doctor at the time was very inquisitive
been hit and miss with other GPs basically based o
Personal experience
"I would say listening to colleagues kind of discus
fact I've had p
Balancing Responsibility and Pressures as a GP
GPs as gatekeepers
"I was diagnosed with tourettiesm which I don't e
the one who's had to go back with the informatio
been in that vicious
Pressure to know more
"My concern with that is that as a GP, I feel press
more about their condition. But I don't have
Responsibility
"well knowledgeable to give a reasonable summa
Inconsistent Availability of Support for Tics Across the Country
Challenges Accessing Support Services
Inconsistent service provision
"the GPs don't actually know who to refer to becau
Lack of intervention
"I think that that causes a barrier in itself because
point in ser
Challenges not exclusive to tics
"But the main problem is actually there's just not
alone absorbing the tics
Referral Complexities and Challenges
Poorly defined referral process
"But neurodevelopmental mental health conditio
that's paediatrics. Then we'll do self-harm and dep
Referral content
"quality of care because they'd already know what
write back and say we can't accept at the
Rejected referrals
"And when referrals are rejected, the patient or ca
which I think is not therapeutic for patients. And I t
because the consultation skills, the languag
Stigma, Dismissal, and Denial of Tics
Stigmatising misconceptions
"If you say Tourette's syndrome, it is usually the k
diagnosis comes in. And actually it is not the case
and people kind of think that that a Tourettes diag
what is the c
Dismissal
"the biggest health misconception is that it's s
something that's a probl
Denial
"The only thing he could have done is say this so
check you out and make sure it isn't". That's the
want you to take it just to find out. If it's not, it
understand what was going on. It was just there

ne tiny, you know, a line of it or something in my years ago medical school. But it's, yeah, definitely not
tured in my initial training or many of my updates that I've had over the years."

't know always what to do with it, where it should go because it hasn't been agreed where it should go
e. We don't have one… We need proper NICE guidelines doing these, telling GPs what to do. GPs look
ody else you know. We follow them to a letter but the kids I see have got multiple diagnosis and mental
problems at the same time. There isn't a NICE guideline that encapsulates what they suffer."

are very sensible through experience, but the difficulty is that a lot of primary care is now delivered by
GPs who are not as familiar with any disorders, let alone tics."

d, I had to go through a process of elimination of what it is not, "let's rule out it's not this". Initially the first
our to explain the movements and different things, so it was just a very, very anxious time and uncertain
time."

n referred to ophthalmology because of eye tics or they've been referred to allergy clinics because of
had all their allergy tests done for lactose intolerance and whatever. The assumption is not that it's a tic
)% of kids have got tics, you know, at some point in their life."

e coming through with Tourettes. I think I was looking at the numbers in [county], it's probably about 200
children in the county."

e. He was curious. He was very pro-wanting to know more and I found that, you know, the treatment has
on the fact of their interest in it. You know if they're like… if they want to do more about it, they'll educate
themselves a lot more about it."

ssing this case on Monday, I think my knowledge is probably greater, but perhaps that's by virtue of the
personal experience. So obviously we kind of read quite a lot into it."

even know as a word. But I think the main thing and the pattern all the way through this is that I've been
n, and it's been up to me to research and educate myself about the condition. Otherwise, I would have
s circle of just taking antipsychotics just to suppress and hold my tics in."

sured by many specialists and many condition groups, charities and people support conditions to learn
a lot of capacity to learn a lot more stuff. But yes, definitely I acknowledge there will be gaps in my
knowledge."

ation of the issue at the start, but of course if things don't settle and don't get better, then you know I'll be
looking for specialist input really."

use the paediatricians won't see, the neurologist won't see and CAMHS won't see, so they don't actually
have anybody to refer to."

e GPs, like us, are frustrated, they're feeling disillusioned. And so there is a sort of, you know, what's the
nding off a referral because it's not going to make any difference"

t enough doctors and specialists out there to cope with the volume of paediatric referrals altogether, let
s on top of it. So the main thing is actually we just don't have the resources."

ns aren't even owned by mental health services because they see it as kind of like physical that "yeah,
pression and anorexia and stuff like that". So this kind of middle ground that no one really knows what to
do with it."

t to refer with, when to refer, and what to put in the referral. So it'll be much less likelihood of us having to
e moment because there's not enough information. So I think that would speed everything up."

arer is not normally copied in this, thus leaving me with the liability… They talk about rejected referrals,
think there's a lot of research to be done about the language we use in our consultations in primary care
ge, the verbal and nonverbal communication are key to whether our interactions are therapeutic."

kind of very rare cases which are portrayed in media and sometimes families are quite worried when the
e. It's a very rare situation when there is coprolalia where they see where they say very obscene words
gnosis it's, it's what they saw on the TV. And then we have to kind of dial it back a bit and tell them about
criteria and how that is quite rare to have such kind of presentation"

severity and I have to wait until it's a severe problem to refer or I can only refer that. I can only refer
blem, you know. It has how you judge impairment and how you judge severity."

ounds like Tourette's, "Hey, I'm just going to make you an appointment, like with a Tourettes clinic just to
only thing because in my head, I was like, "this isn't Tourettes"... If has said "there's test for Tourettes, I
t's not. If it is, it is". I would have taken it like, you know, I was a pretty sort of desperate situation to
e was, I don't know if there was. He didn't refer me to anywhere. He just left it as, "if it gets worse come

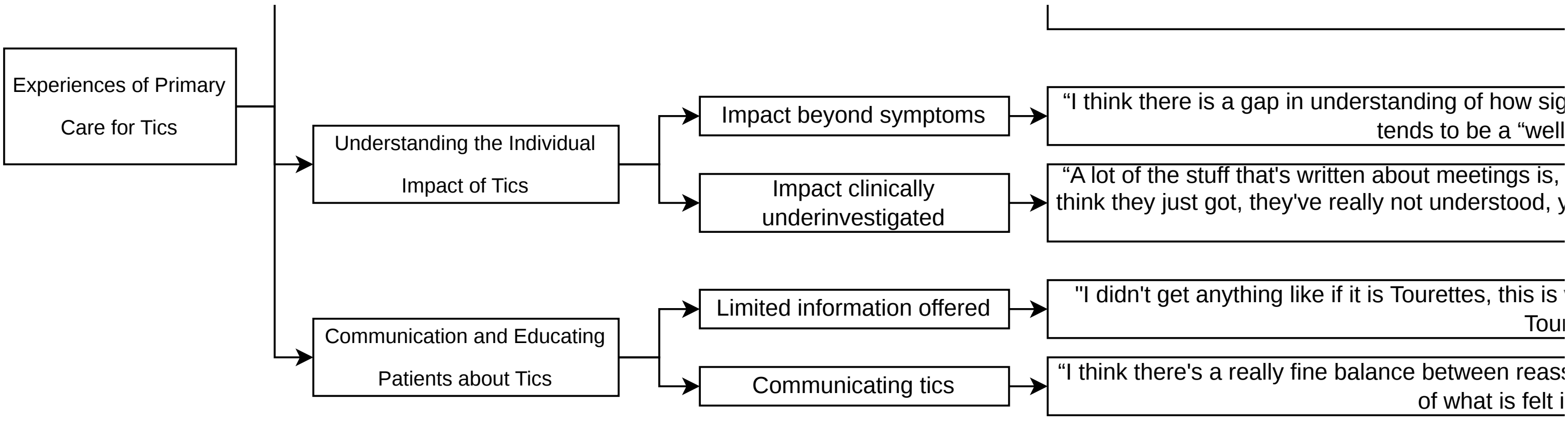
Experiences of Primary Care for Tics
Understanding the Individual Impact of Tics
Impact beyond symptoms
"I think there is a gap in understanding of how sig
tends to be a "well
Impact clinically underinvestigated
"A lot of the stuff that's written about meetings is,
think they just got, they've really not understood, y
Communication and Educating Patients about Tics
Limited information offered
"I didn't get anything like if it is Tourettes, this is
Tour
Communicating tics
"I think there's a really fine balance between reas
of what is felt i

| |
|---|
| back". And that was it." |

| |
|---|
| gnificant tics can be in terms of pain, mental health difficulties and the impact on daily life. I think there's l, there's little movement or a little sound. What's the concern about it?"." |
| "oh, they're doing well", "they're fine", "they're OK", and "they're dealing well with their tics". And I often you know, in that 15-20 minute or 10-minute GP appointment. It's not really understood what is going on for a family at all" |

| |
|---|
| what you know will happen or this is what will be done or with or kind of any explanation really around rettes? There was none of that. It was just, "I'll refer you"." |
| suring people and people feeling not heard and dismissed. And I know in the Tourettes community, a lot is that they're being dismissed. And I can see there's a big disparity." |
