# Supplementary material for: Growing Proficiency in Tic Disorder Care and Skills (GP-TiCS): a thematic analysis of patient and professional group experiences in primary care for primary tic disorders
Source: BMC Prim Care. 2026 Jul 30;27:388. doi: 10.1186/s12875-026-03496-y (PMC13711887; doi:10.1186/s12875-026-03496-y)
Supplement: Supplementary file 7 [file 12875_2026_3496_MOESM7_ESM.pdf]

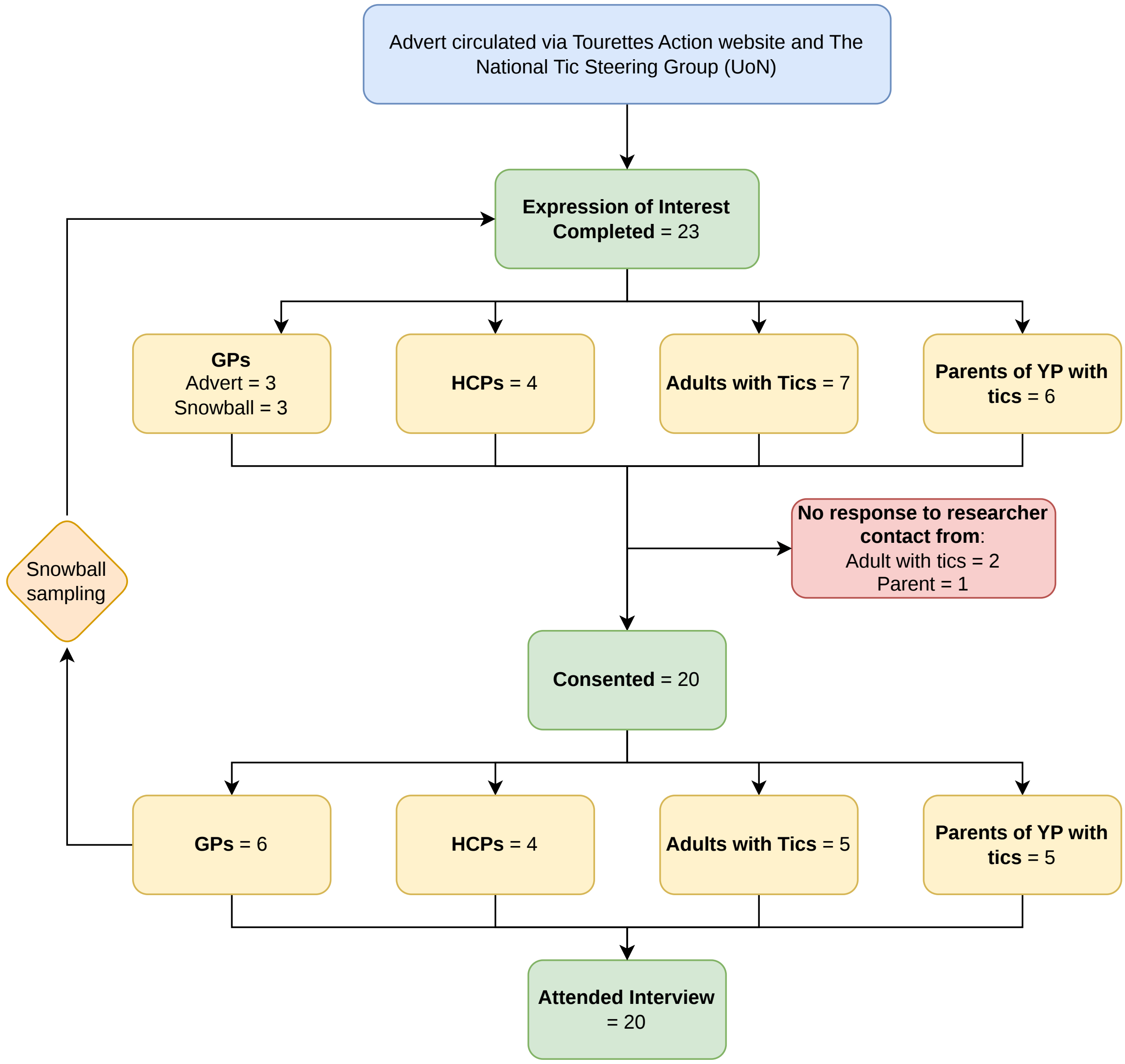
Advert circulated via Tourettes Action website and The National Tic Steering Group (UoN)
Expression of Interest Completed = 23
GPs Advert = 3 Snowball = 3
HCPs = 4
Adults with Tics = 7
Parents of YP with tics = 6
No response to researcher contact from: Adult with tics = 2 Parent = 1
Snowball sampling
Consented = 20
GPs = 6
HCPs = 4
Adults with Tics = 5
Parents of YP with tics = 5
Attended Interview = 20
