# Supplementary material for: Growing Proficiency in Tic Disorder Care and Skills (GP-TiCS): a thematic analysis of patient and professional group experiences in primary care for primary tic disorders
Source: BMC Prim Care. 2026 Jul 30;27:388. doi: 10.1186/s12875-026-03496-y (PMC13711887; doi:10.1186/s12875-026-03496-y)
Supplement: Supplementary file 8 [file 12875_2026_3496_MOESM8_ESM.pdf]

Additional File 8

Summary of themes and sub-themes.

Theme	Description	Subtheme	Exemplar
Factors Impacting GPs' Knowledge and Education of Tics	This theme explores how GPs receive little education on tics, with few guidelines available. This can lead to overcomplicated clinical investigations. GPs often feel under pressure to be more knowledgeable about many disorders, but question whether this is their responsibility.	Limited Commissioned Training Opportunities and Guidelines<br>Everything but a Tic Disorder<br>Individualised Barriers and Facilitators of Tic Knowledge<br>Balancing Responsibility and Pressures as a GP	“I think the main thing and the pattern all the way through this is that I've been the one who's had to go back with the information, and it's been up to me to research and educate myself about the condition”
Inconsistent Availability of Support for Tics Across the Country	This theme discusses how access to appropriate support for tics varies nationally and factors which compound referrals include insufficient evidence and only referring once symptoms worsen.	Challenges Accessing Support Services<br>Referral Complexities and Challenges	“the GPs don't actually know who to refer to because the paediatricians won't see, the neurologist won't see and CAMHS won't see, so they don't actually have anybody to refer to.”
Experiences of Primary Care for Tics	This theme explores how patient experiences varied in primary care based on factors including: understanding of the impact of tics, the communication between patient and clinicians, and tic-related stigma.	Stigma, Dismissal, and Denial of Tics<br>Understanding the Individual Impact of Tics<br>Communication and Educating Patients about Tics	"they're OK", and "they're dealing well with their tics". And I often think they just got, they've really not understood, you know, in that 15-20 minute or 10-minute GP appointment. It's not really understood what is going on for a family at all”.
