# Supplementary material for: Growing Proficiency in Tic Disorder Care and Skills (GP-TiCS): a thematic analysis of patient and professional group experiences in primary care for primary tic disorders
Source: BMC Prim Care. 2026 Jul 30;27:388. doi: 10.1186/s12875-026-03496-y (PMC13711887; doi:10.1186/s12875-026-03496-y)
Supplement: Supplementary file 9 [file 12875_2026_3496_MOESM9_ESM.pdf]

Additional File 9

Simplified comparison table of studies included in Parker at al. (2024) systematic review

Reference	Measure	Sample	Summary of findings
**Qualitative Studies**			
Ludlow, Brown & Schulz (2018),<br>*UK*	Semi-structured interviews	15 parents/caregivers	Parents reported difficulties managing tics; limited professional knowledge; stigma; limited school support; and inadequate healthcare guidance.
Rivera-Navarro, Cubo & Almanzan (2009),<br>*Spain*	Focus groups	29 participants (CYP, adults, caregivers, HCPs)	Diagnosis delays; limited understanding by professionals; stigma; and communication difficulties between families and healthcare services.
**Quantitative Studies**			
Alalwan (2022),<br>*Saudi Arabia*	Survey	59 HCPs and 316 medical students	Moderate knowledge of tic disorders; family physicians performed better; limited awareness of behavioural therapies.
Ganos et al. (2021),<br>*International*	Survey	346 Movement Disorder Society clinicians	Diagnosis mainly based on clinical assessment; behavioural therapy preferred but access limited; family education viewed as essential.
Katona (2013),<br>*UK*	Survey	94 medical students	Limited understanding of tic causes and evidence of persistent stigma despite increasing awareness.

Marcks et al. (2004), *USA*	Survey	37 secondary HCPs, 30 primary HCPs	Generally good knowledge but poor understanding of diagnostic criteria and behavioural interventions; awareness of stigma was high.
O'Hare (2016), *Australia*	Survey	86 caregivers of CYP with tics, 104 caregiver control group	Frequent dissatisfaction with healthcare and school support; misdiagnosis and stigma remained common.
Wand et al. (1992), *Canada*	Survey	462 respondents with personal or familial experience of tics	Misdiagnosis common; most participants relied on non-medical sources for information, e.g., media.
Wellen et al. (2023), *USA*	Survey	186 parents/caregivers	Families consulted multiple providers; limited specialist access; financial barriers; and primary care knowledge gaps were common.
**Mixed Method Studies**			
Cuenca et al. (2015), *UK*	Survey & interviews	295 caregivers; 42 CYP	Parents reported limited professional knowledge; referral difficulties; and limited access to specialist services.
Marino et al. (2023), *UK*	Survey (open & closed questions)	33 adults with tics and 94 parents of CYP with tics	Primary care frequently failed to recognise tics; referrals were delayed; and many families were dissatisfied with support.
Rodin et al. (2021), *Uganda*	Survey & interviews	138 secondary care HCPs and 14 students;<br>6 HCPs interviewed	Limited knowledge and confidence diagnosing tic disorders; cultural beliefs contributed to stigma and misdiagnosis; strong demand for education.
Stacy et al. (2023), *USA*	Survey	300 secondary care HCPs and 67 parents/caregivers	Inconsistent knowledge of diagnosis and treatment among clinicians; communication and recognition of stigma varied across professionals.
